# Platelet-Related Signature in Colorectal Cancer: Prognosis and Therapy Guidance

**DOI:** 10.3390/ijms27188312

**Published:** 2026-09-18

**Authors:** Yun Xie, Jun Li, Zuwei Yan, Wenguang Zhang

**Affiliations:** 1 Faculty of Science, Inner Mongolia Agricultural University, Hohhot 010018, China; xieyunhust@163.com; 2 School of Life Science and Technology, Inner Mongolia University of Science and Technology, Baotou 014010, China; 3 Inner Mongolia Engineering Research Center of Genomic Big Data for Agriculture, Inner Mongolia Agricultural University, Hohhot 010018, China; 4 Institute for Rural Revitalization, Inner Mongolia Agricultural University, Hohhot 010018, China

**Keywords:** colorectal cancer, platelet-related gene signature, prognostic stratification, immune microenvironment, drug sensitivity association

## Abstract

Platelets are key components of the tumor microenvironment that contribute to colorectal cancer (CRC) progression. However, effective platelet-related prognostic tools for CRC risk stratification and prognostic assessment are lacking. Therefore, we aimed to develop a platelet-related prognostic model to improve risk stratification and prognostic evaluation. A tumor-cell-expressed platelet-related transcriptional signature, termed platelet-related risk score (PLRS), was established using LASSO and Cox regression based on TCGA-COADREAD, GSE39582 and GSE183635. After strict screening for intact survival information, 573, 531 and 85 tumor samples were retained correspondingly. Patients were stratified into high- and low-PLRS groups according to the median cutoff value of PLRS derived from the training cohort. A single-cell dataset GSE178341 was utilized for single-cell transcriptomic validation. All analyses were performed in R. Wilcoxon test, Kaplan–Meier method, log-rank test, multivariate Cox regression and ROC curves were applied with two-sided *p* < 0.05. qRT-PCR was conducted for hub gene validation. The PLRS model involved six prognostic genes (*TIMP1*, *TTYH3*, *GPX4*, *PLCB4*, *CA2*, and *LITAF*). The high- and low-PLRS groups showed significant differences in disease stage and survival in TCGA datasets, which was confirmed in the GEO dataset (GSE39582). The multivariate analysis established the PLRS as an independent prognostic factor. Single-cell analyses revealed distinct transcriptional trajectories, immune microenvironments, and communication patterns between the PLRS groups. Stromal, immune, and ESTIMATE scores positively correlated with PLRS, whereas tumor purity was inversely correlated. Exploratory drug sensitivity analysis using GDSC and CCLE cell line datasets suggested potential associations between PLRS and multiple therapeutic agents, warranting validation in clinical cohorts. qRT-PCR in RKO cells compared with the CCD-18Co fibroblast control partially confirmed the expression trends of four PLRS genes (*TIMP1*, *GPX4*, *LITAF*, and *PLCB4*), whereas *TTYH3* showed no significant difference and *CA2* was not tested due to primer-design failure. Validation in normal colonic epithelial cells is warranted. The PLRS model demonstrated statistically significant prognostic value and may serve as a complementary tool for CRC risk stratification, providing a basis for further investigation into platelet-related transcriptional programs.

## 1. Introduction

Colorectal cancer (CRC) is the third leading cause of cancer-related deaths worldwide. According to the International Agency for Research on Cancer, approximately 1,932,000 new CRC cases were diagnosed worldwide in 2020, with 935,000 annual deaths [1]. CRC is a heterogeneous malignancy that primarily originates from adenomatous polyps or adenomas [2]. In addition to the adenoma–carcinoma sequence, CRC can occur via a serrated pathway [3]. The risk of CRC is influenced by factors such as age, sex, environment, genetics, lifestyle, nutritional status, and physical activity [4]. The treatment options for CRC typically include surgical resection, radiotherapy, chemotherapy, and targeted therapy [5]. Owing to the low rates of early screening and diagnosis, most patients are diagnosed at advanced stages [6] and recurrence rates are high, with some patients experiencing relapse and metastasis even after surgery [7]. The efficacy of CRC drug treatments is suboptimal, with low sensitivity to chemotherapy and radiotherapy. Additionally, surgical treatment is challenging, particularly for advanced-stage patients whose surgical resection success rates are low, and postoperative survival is poor [8]. Despite the advances in surgery and chemoradiotherapy, robust prognostic biomarkers and therapeutic targets for CRC are urgently required.

Platelets are tiny cell fragments in the blood that play key roles in blood coagulation and hemostasis. Platelet and coagulation system activation have been linked to cancer progression, making them a new avenue for CRC prognostic research. Activated platelets interact with tumor cells to promote invasion, migration, and angiogenesis [9]. In circulation, platelets shield tumor cells from immune elimination and facilitate their arrest at endothelial sites, contributing to secondary lesion development [10]. Platelets promote tumor metastasis by binding to circulating tumor cells [11]. They also enhance bone marrow-derived mesenchymal stem cell activity in tumor metastasis [12]. Platelets are increasingly recognized not only as key effectors of tumor progression but also as promising sources for liquid biopsy biomarkers, owing to their active crosstalk with tumor cells and their capacity to modulate immune responses [13]. Therefore, platelet-related genes (PL-genes) can facilitate disease diagnosis [14]. CRC PL-genes may provide biomarkers or therapeutic targets for modulating tumor immunity [15]. However, the association between PL-genes and CRC prognosis is unclear.

Prognostic signature genes are vital in precision cancer medicine. By analyzing gene expression in tumor tissues, we can identify biological features associated with tumor prognosis, which may aid risk assessment. However, to the best of our knowledge, effective platelet-related prognostic tools for CRC risk stratification are lacking.

In this study, we aimed to improve prognostic stratification for patients with colorectal cancer by constructing a platelet-related risk score (PLRS), defined as a transcriptional signature based on platelet-associated gene sets expressed in tumor cells, which may partially reflect platelet–tumor crosstalk. This model may serve as a complementary tool for clinical risk assessment and provide a basis for further investigation into platelet-related transcriptional programs in CRC.

## 2. Results

### 2.1. Dysregulation of Platelet-Related Factors in CRC

#### 2.1.1. Screening Meaningful Platelet-Related Genes

Based on GSE183635 and TCGA datasets comprising CRC and normal control samples, using a screening threshold of *P_adj_* < 0.05 and |log_2_FoldChange| > 0.58, we identified 665 and 202 PL-genes significantly overexpressed in normal and CRC samples, respectively (Supplementary Figure S1A,B). In TCGA dataset, 6962 genes were significantly overexpressed in normal samples, whereas 12,173 were significantly overexpressed in CRC samples (Supplementary Figure S2A,B). To obtain meaningful PL-genes, we intersected the DEGs between normal and CRC samples with the differentially expressed PL-genes. Thirty-seven platelet genes were overexpressed and 113 were underexpressed in CRC (Supplementary Figure S2C,D). These 150 genes represent the selected PL-genes.

#### 2.1.2. Univariate Cox Regression Analysis for Screening Prognostic Platelet Marker

**Figure 1:**
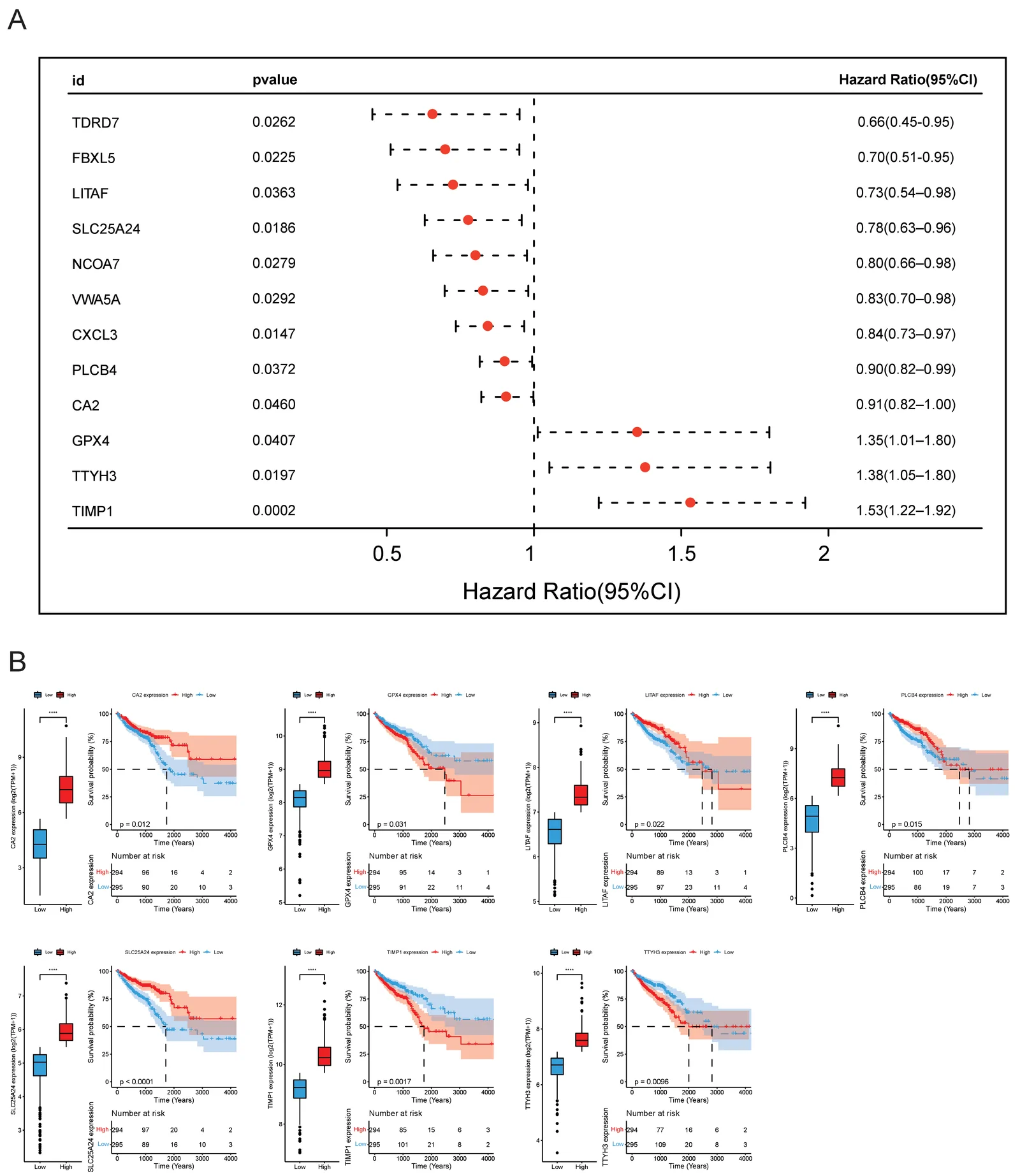
Prognostic-associated platelet-related factors. (**A**) Twelve platelet-related factors with significant prognostic differences. (**B**) Seven platelet-related factors with significant prognostic differences in both high-expression and low-expression groups. **** *p* < 0.0001.

Based on the aforementioned 150 PL-genes, we conducted univariate Cox regression analysis to assess their prognostic relevance. This analysis identified 12 genes with significant prognostic differences, among which *GPX4*, *TIMP1*, and *TTYH3* exhibited hazard ratios greater than 1 (Figure 1A). Using the median gene expression as a cutoff, we stratified the patients into high- and low-expression groups. Of the 12 genes with significant prognostic differences, only seven PL-genes demonstrated significant prognostic differences between the high- and low-expression groups (Figure 1B).

### 2.2. Construction and Validation of a Platelet-Related Prognosis Model for CRC

#### 2.2.1. Machine Learning-Based Construction of PLRS Model

**Figure 2:**
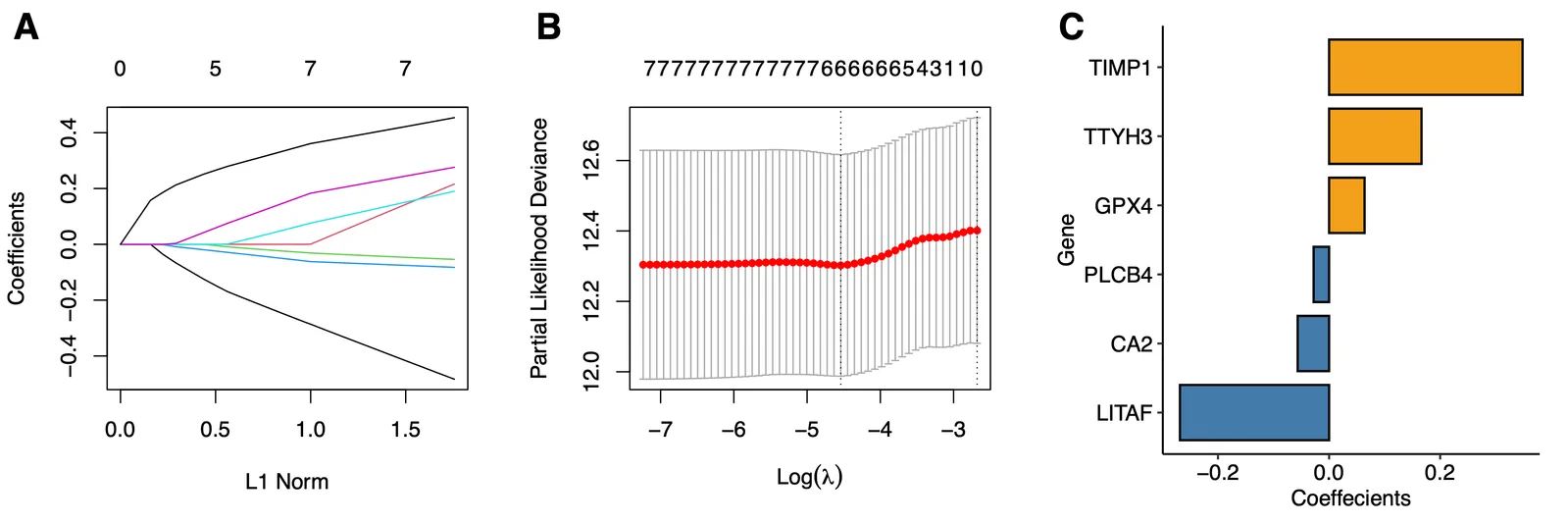
Construction of the prognostic model using LASSO regression. (**A**) The LASSO coefficient profiles of candidate platelet-related genes. (**B**) A partial likelihood deviance plot for cross-validation. (**C**) A forest plot showing the regression coefficients of the six genes included in the final prognostic model.

LASSO regression analysis revealed that six of the seven PL-genes had significant prognostic relevance. We constructed the PLRScore based on the sum of the products of the LASSO regression coefficients and gene expression levels. This model included six genes (*TIMP1*, *LITAF*, *TTYH3*, *GPX4*, *CA2*, and *PLCB4*) that are referred to as feature genes in the PLRS model. Figure 2 shows the relevant parameters of the LASSO model and LASSO coefficients of the six genes.

PLRScore=0.3485×expression of TIMP1+0.1669×expression of TTYH3+0.069×expression of GPX4−0.2691×expression of LITAF−0.0567×expression of CA2−0.0283×expression of PLCB4

#### 2.2.2. Univariate and Multivariate Cox Analysis Confirming PLRS as an Independent Prognostic Factor

Using both univariate and multivariate Cox analyses, we evaluated the prognostic performance of various clinical factors along with the PLRS. In both analyses the PLRS demonstrated significant prognostic efficacy in the TCGA-COADREAD and GSE39582 datasets, and was established as an independent prognostic factor (Supplementary Figure S3).

#### 2.2.3. Single-Cell Transcriptomic Analysis Reveals Platelet-Related Patterns

After completing data preprocessing, we first used the “SingleR” package (v2.0.0) to annotate the cell types. In total, 121,710 epithelial cells were obtained. Based on these epithelial cells, we used the “copykat” function to identify malignant epithelial cells with non-integer CNVs, resulting in 46,915 malignant epithelial cell lines.

**Figure 3:**
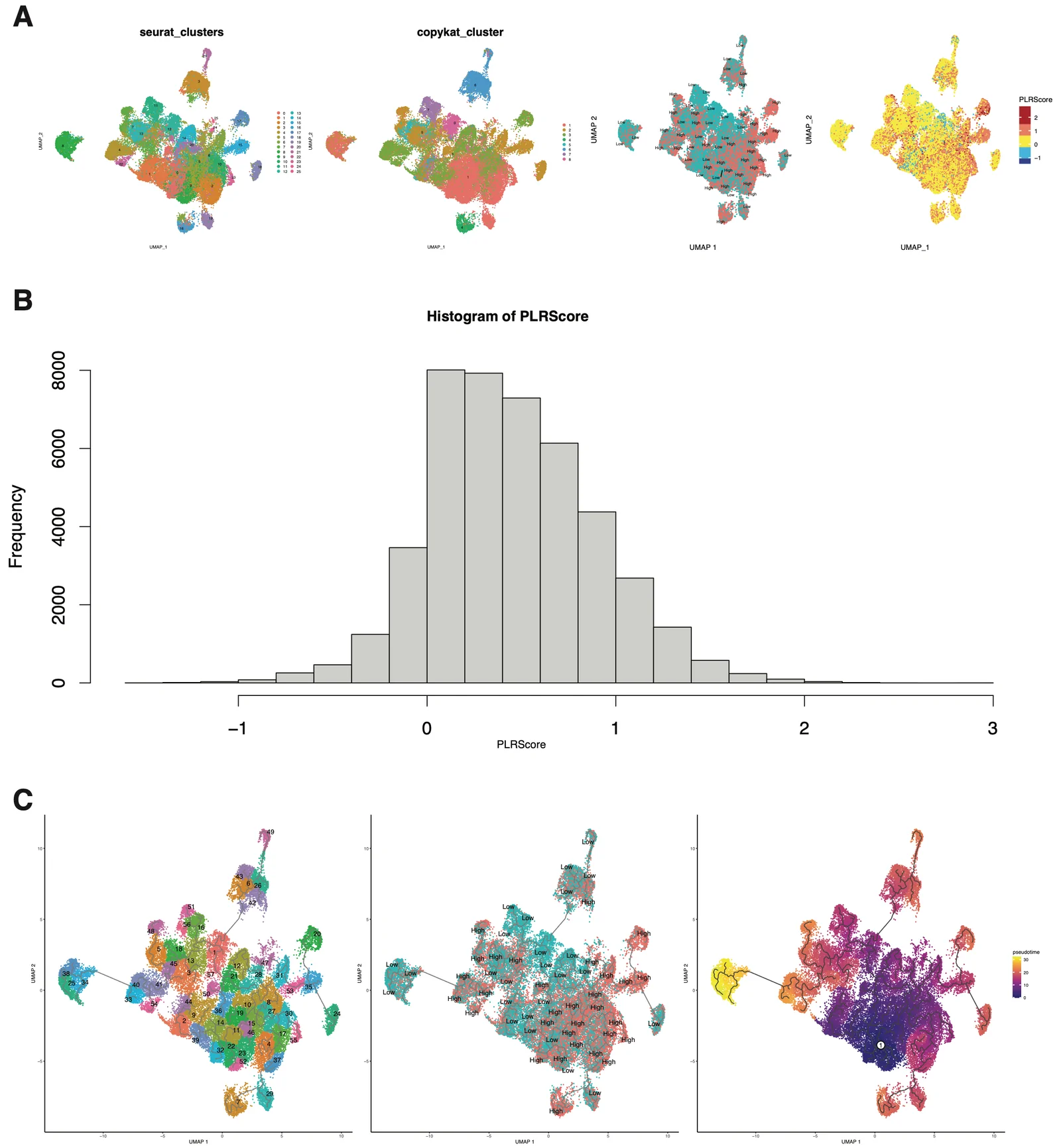
Single-cell clustering and PLRS pseudotime analysis. (**A**) Subgroups of tumor cells, copy number variation-based subgroups, distribution of PLRScore in tumor cells, and PLRS grouping. (**B**) Frequency distribution of PLRScore in tumor cells. (**C**) Pseudotime trajectory of tumor cells. PLRS, platelet risk score.

Owing to the nature of single-cell sequencing, there was considerable variability in the genes detected within individual cells. We focused on the cells in which at least one of the six feature genes was measured. After analysis, we found that of 46,915 malignant epithelial cells (tumor cells), 44,353 cells had sequencing results for at least one of the six feature genes. Using the “Seurat” package (v4.2.0), we performed clustering analysis on these 44,353 malignant epithelial cells and identified 25 tumor cell subpopulations (Figure 3A). We applied the PLRS calculation method to the single-cell samples. The cells were then categorized into high- and low-PLRS groups based on the median single-cell PLRScore (Figure 3B). Pseudotime analysis using the “monocle3” package (v1.3.1) revealed a transcriptional continuum from high-PLRS to low-PLRS tumor cells (Figure 3C), indicating that PLRS status is associated with cell-state heterogeneity along the inferred trajectory.

#### 2.2.4. PLRS and Immune Microenvironment

**Figure 4:**
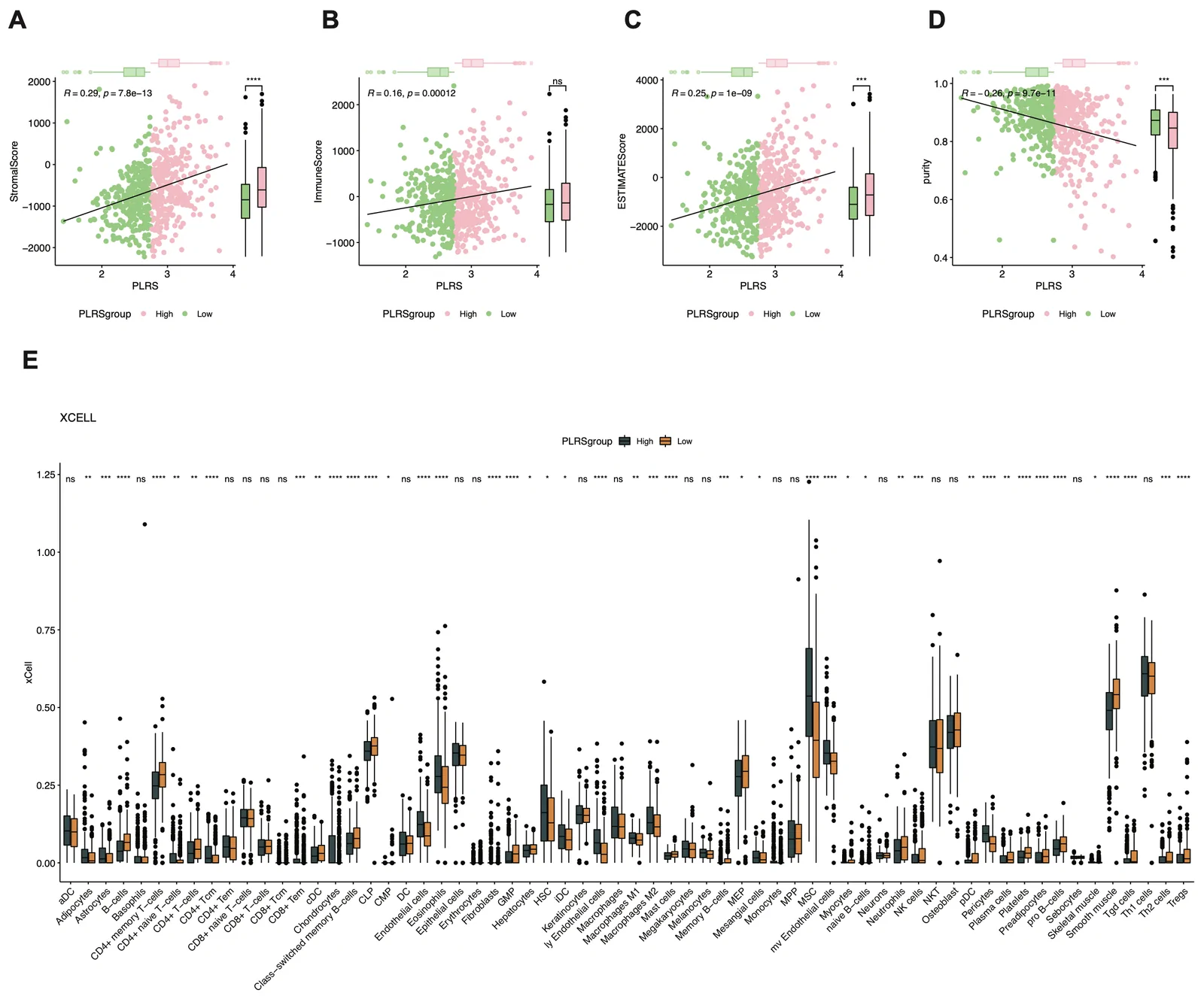
Immunological landscape between high- and low-PLRS groups. (**A**–**D**) Correlation between PLRS and tumor stromal score, immune score, estimate score, and tumor purity. (**E**) Immune cell infiltration analysis based on ssGSEA. PLRS, platelet risk score. * *p* < 0.05, ** *p* < 0.01, *** *p* < 0.001 and **** *p* < 0.0001.

Immunocell infiltration analysis based on the xCell algorithm revealed significant differences in the infiltration proportions of most immune cells between the high- and low-PLRS groups, with the high-PLRS group showing significantly higher levels than those of the low-PLRS group (Figure 4E). Similar analyses using the ssGSEA and Cibersort algorithms yielded results that were highly consistent with those obtained using the xCell algorithm (Supplementary Figure S4). Further analysis using the “estimate” package (v1.0.13) to assess the stromal score, immune score, ESTIMATE score, and tumor purity revealed significant positive correlations between the stromal, immune, and ESTIMATE scores with PLRSore (Figure 4A–C), whereas tumor purity showed a significant negative correlation with PLRSore (Figure 4D).

#### 2.2.5. Differences in Cell–Cell Communication Between High- and Low-PLRS Groups

**Figure 5:**
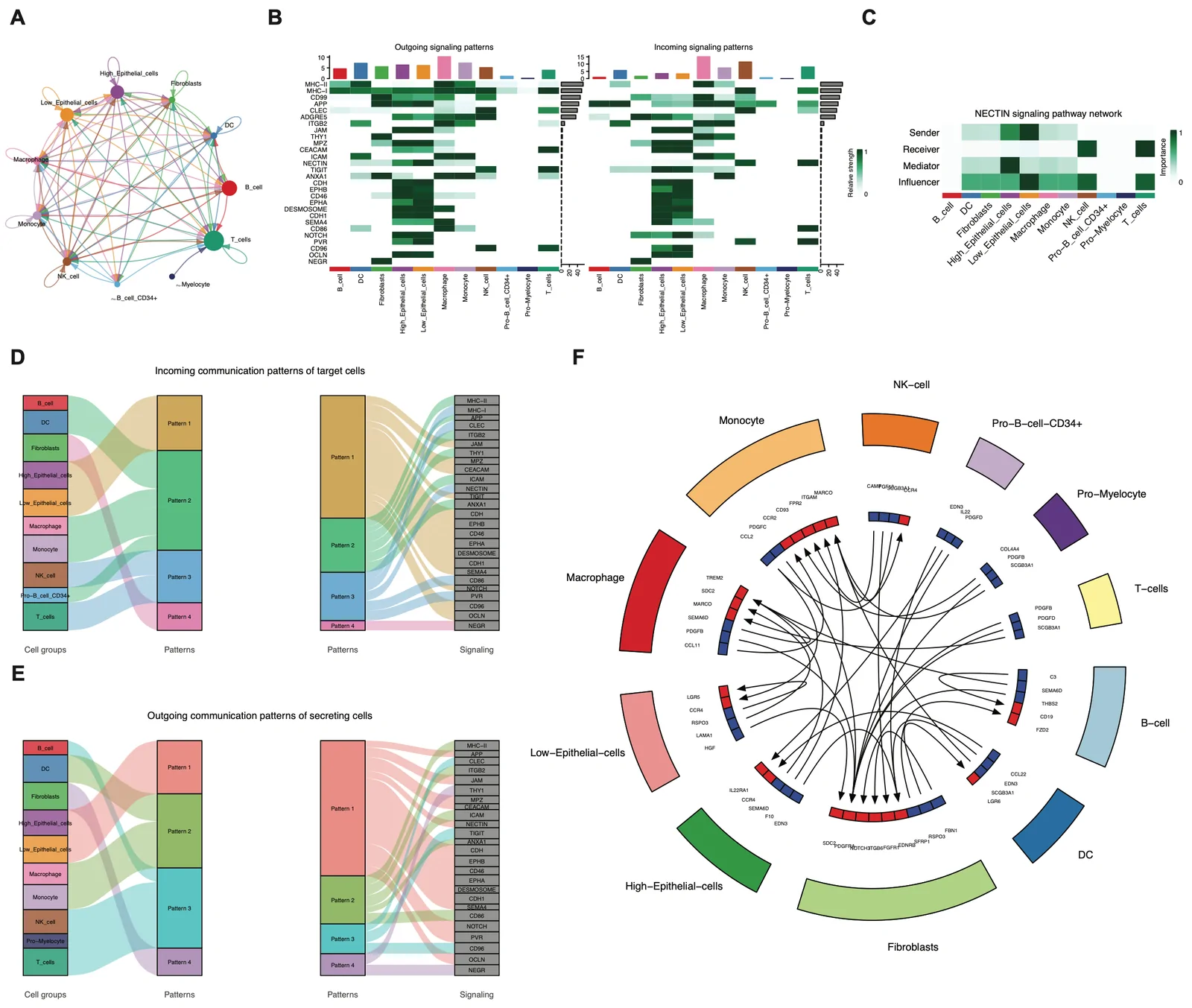
Specific cell–cell communication between high- and low-PLRS cells. (**A**–**E**) Communication patterns between cells. (**F**) Ligand–receptor pairs between cells.

Based on single-cell data, we first analyzed the communication patterns between tumor epithelial and immune cells using the “CellChat” package (v1.1.3) (Figure 5A). The analysis revealed distinct communication patterns between cells from the high- and low-PLRS groups and immune cells in both the incoming and outgoing directions. Specifically, in the outgoing direction, significant differences in communication strength were observed between the high- and low-PLRS groups for the NECTIN, PVR, and EPHA pathways. In the upstream direction, notable differences were observed in the EPHB, SEMA4, CD96, NOTCH, and ADGRE5 pathways (Figure 5B). The signaling pathway networks exhibited variations in senders, mediators, and influencers between cells in the high- and low-PLRS groups (Figure 5C). The communication patterns between tumor and immune cells, along with the corresponding signaling pathways for the different patterns, are illustrated in Figure 5D,E.

Further analysis using the “CellTalker” package identified ligand–receptor pairs that differed between high- and low-PLRS cells, and specific immune cells. Specifically, both high- and low-PLRS cells exhibited mutual communication with macrophages, monocytes, and fibroblasts. Additionally, high-PLRS cells uniquely communicated with dendritic and Pro-B-cell-CD34+ cells (Figure 5F).

### 2.3. Differences in Chemotherapy Drug Resistance Between High- and Low-PLRS Groups

**Figure 6:**
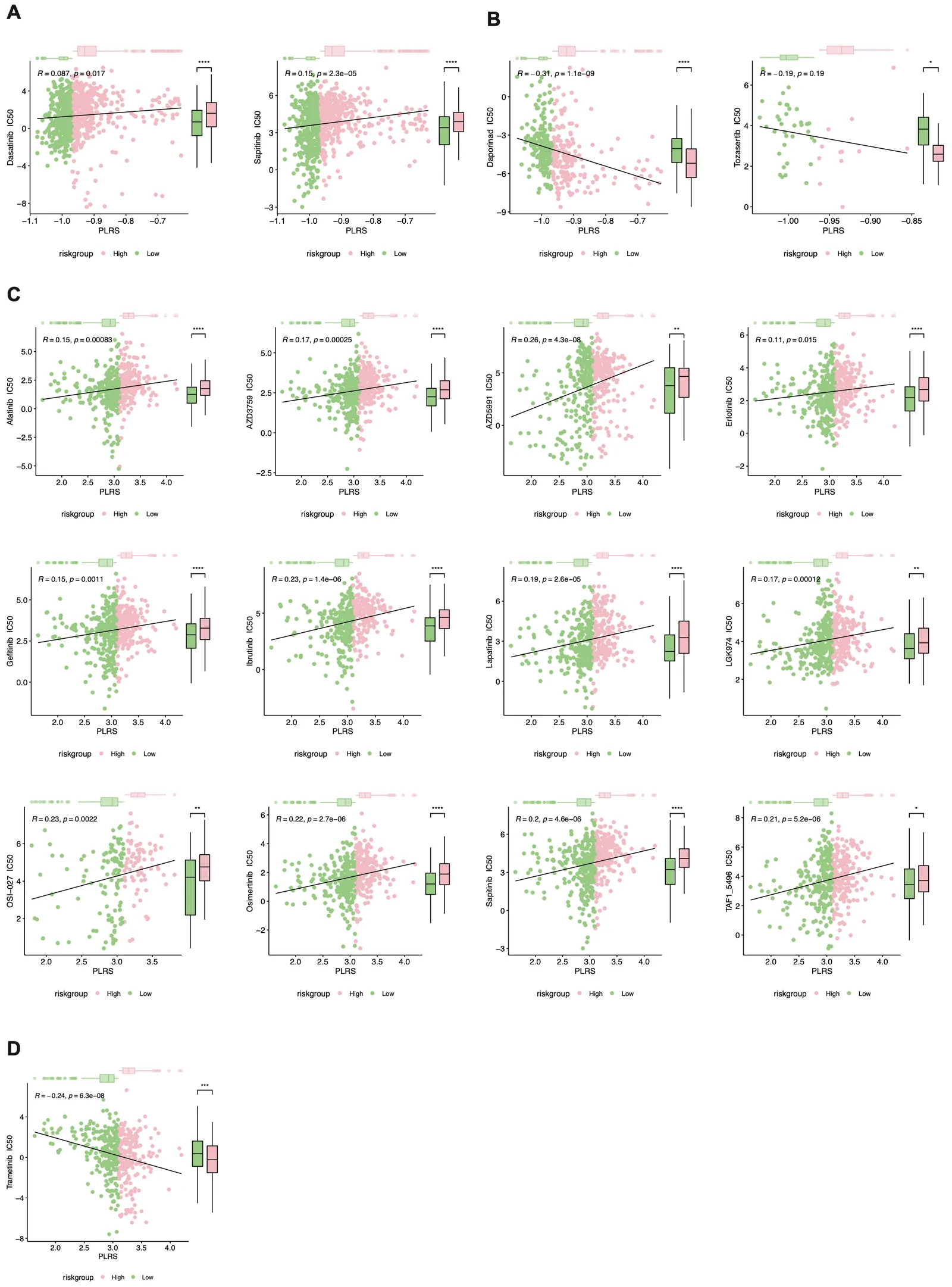
Exploration of PLRS-related drugs in the GDSC and CCLE databases. (**A**) Drugs in the GDSC database showing a positive correlation between IC50 and PLRS values. (**B**) Drugs in the GDSC database showing a negative correlation between IC50 and PLRS values. (**C**) Drugs in the CCLE database showing a positive correlation between IC50 and PLRS values. (**D**) Drugs in the CCLE database showing a negative correlation between IC50 and PLRS values. CCLE, the Cancer Cell Line Encyclopedia; PLRS, platelet risk score. * *p* < 0.05; ** *p* < 0.0; *** *p* < 0.001; **** *p* < 0.0001.

Further analyses were conducted to investigate the differences in chemotherapy drug resistance between the high- and low-PLRS groups. In the GDSC database, the IC50 values for Dasatinib and Sapitinib positively correlated with the PLRS, and there were significant differences in the IC50 values between the PLRS groups (Figure 6A). Conversely, the IC50 values for daporinad and tozasertib were negatively correlated with the PLRS, with significant differences in the IC50 observed between the groups (Figure 6B).

In the CCLE dataset, we identified 12 drugs (Afatinib, AZD3759, AZD5991, Erlotinib, LGK974, Lapatinib, Ibrutinib, Gefitinib, OSI-027, Osimertinib, Sapitinib, and TAF1-5496) with IC50 values that were significantly and positively correlated with the PLRS (Figure 6C), whereas the IC50 of trametinib was significantly and negatively correlated with PLRS (Figure 6D). The IC50 values of these drugs significantly differed between the high- and low-PLRS groups. Notably, in both the CCLE and GDSC datasets, the IC50 values of the drugs were positively correlated with PLRS and significantly higher in the high-PLRS group than those in the low-PLRS group, suggesting that high-PLRS cells may exhibit relatively higher resistance to certain drugs in cell line models. However, these findings are based on cell line databases and do not directly establish clinical drug response; validation in patient cohorts is warranted.

### 2.4. Experimental Validation of Feature Gene Expression in Platelet-Related Scoring Models in CRC

**Figure 7:**
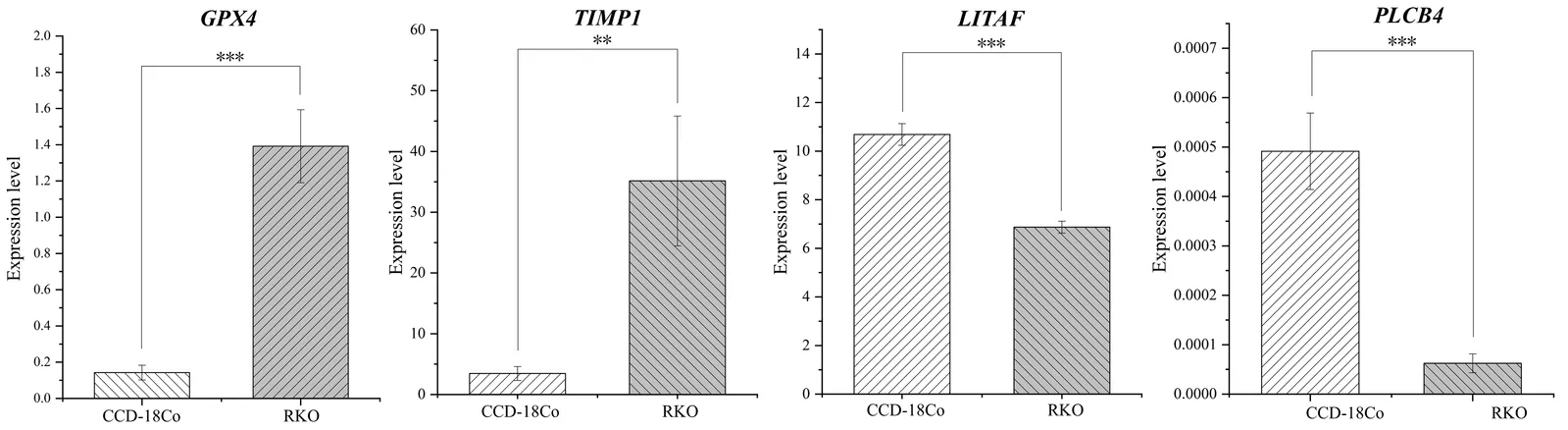
Relative expression levels of PLRS signature genes in CCD-18Co and RKO cell lines. Data are presented as mean ± standard deviation. Differences between groups were analyzed using Student’s test. (** *p* < 0.01 and *** *p* < 0.001).

In the RKO CRC and CCD-18Co normal colonic fibroblast cell line, the expression levels of *TIMP1*, *GPX4*, *LITAF, PLCB4*, and *TTYH3* from the PLRS model were examined (note: due to unsuccessful specific primer design for *CA2* after multiple attempts, *CA2* was not investigated further at the molecular level in this study). *TIMP1* and *GPX4* mRNA were significantly upregulated (*p* < 0.01) in the RKO cell line, whereas *LITAF* and *PLCB4* were significantly downregulated (*p* < 0.001); *TTYH3* showed no significant difference (Figure 7). Despite these limitations, the expression trends of four of the five detectable genes (*TIMP1*, *GPX4*, *LITAF*, and *PLCB4*) were consistent with the direction of their coefficients in the PLRS model, providing partial experimental support for the model’s gene signature. However, we acknowledge that the lack of validation for *CA2* and the non-significant result for *TTYH3* indicate that this experimental verification is incomplete. Further validation across additional CRC cell lines and clinical samples is warranted to comprehensively confirm the six-gene signature.

## 3. Discussion

We developed a PLRS model based on bulk and single-cell RNA-seq data, which demonstrated statistically significant prognostic value in CRC. Exploratory analyses also suggested potential associations with immunotherapy outcomes and drug sensitivity. The PLRS signature may serve as a complementary tool for risk stratification and provide a basis for further investigation into platelet-related transcriptional programs and potential therapeutic strategies, although prospective validation is warranted before any clinical application.

We identified six platelet-related genes (*CA2*, *GPX4*, *LITAF*, *PLCB4*, *TIMP1*, and *TTYH3*) with significant differences in expression between the high- and low-PLRS groups, and their expression patterns were validated using molecular experiments. Notably, the direction of the coefficient in the PLRS model was consistent with these changes in expression, confirming the accuracy of the model for capturing CRC-specific PL-gene signatures. *CA2* promotes proliferation and tumor growth in multiple cancer types [16]; its dysregulation in the PLRS model may contribute to CRC progression by enhancing tumor cell viability. *GPX4* is overexpressed in CRC and serves as an indicator of immune cell infiltration in tumor tissues [17]. The combination of sorafenib and *GPX4* inhibitors has been proposed as a promising therapeutic strategy for CRC [18]; therefore, *GPX4* in the PLRS model can guide the selection of targeted therapy. Both in vivo and in vitro experiments have confirmed that *LITAF* inhibits CRC cell proliferation, migration, and invasion, and induces apoptosis [19]. Its downregulation in CRC (consistent with our molecular data) contributes to the prognostic efficacy of the PLRS model, highlighting its potential as a core component of platelet-related prognostic stratification and a candidate therapeutic target.

Oxaliplatin (OXA) is a first-line chemotherapeutic agent for metastatic CRC and *PLCB4*, an OXA resistance-related gene, is a potential therapeutic target in OXA-resistant cases [20]. Previous studies have reported upregulated *PLCB4* expression in CRC tissues [21] and its prognostic value [22]; however, we observed downregulated *PLCB4* in CRC samples. This discrepancy may reflect subtype-specific expression patterns or tissue heterogeneity, further underscoring the necessity to integrate multigene signatures in the PLRS model to overcome the limitations of single-gene prognostic markers. *TIMP1* promotes colon cancer tumorigenesis and metastasis [23], and *TTYH3* has been identified as a potential prognostic and diagnostic biomarker for CRC [24]. Their inclusion in the PLRS model enhances the comprehensiveness of platelet-related prognostic stratification because these genes collectively reflect the multifaceted role of platelets in CRC progression.

The univariate and multivariate Cox analyses showed that PLRS was an independent prognostic factor for CRC in both TCGA-COADREAD and GSE39582 datasets, indicating consistent prognostic value across different CRC cohorts. Its independence from traditional clinical factors (e.g., disease stage) suggests additional value of the PLRS model in refining risk stratification in CRC, potentially identifying high-risk patients even within the same clinical stage. This may help inform risk-adapted surveillance or treatment strategies, although prospective validation is needed before clinical implementation.

Single-cell transcriptome analysis of CRC malignant epithelial cells revealed an inferred transcriptional continuum from high-PLRS to low-PLRS cell populations, suggesting that high-PLRS cells may occupy a transcriptional state associated with stemness-related programs. Clinically, this finding offers a potential biological rationale for the poor prognosis of patients with high PLRSs in CRC; however, pseudotime analysis is a computational inference and does not establish temporal progression or regenerative capacity. Further functional studies are needed to determine whether high-PLRS cell populations could represent candidate therapeutic targets for high-risk CRC patients identified by the PLRS model.

Pseudotime analysis revealed a transcriptional continuum from high-PLRS to low-PLRS cell populations, suggesting that PLRS status is associated with cell-state heterogeneity along the inferred trajectory. This finding offers a potential biological rationale for the poor prognosis observed in patients with high PLRS, as the transcriptional features of high-PLRS cells may reflect enhanced proliferative or stemness-associated programs that could contribute to tumor recurrence and treatment resistance. However, as pseudotime is a computational inference based on transcriptomic similarity rather than a measure of chronological progression, these observations should be interpreted with caution. Further functional experiments and lineage-tracing studies are warranted to determine whether targeting high-PLRS cell populations could confer therapeutic benefit in high-risk patients identified by the PLRS model.

Immune cell infiltration analysis showed significantly higher infiltration proportions in the high-PLRS group, with positive correlations between PLRS and stromal, immune, and ESTIMATE scores, and an inverse correlation with tumor purity. These findings align with the concept that platelets actively shape the immune landscape [25], supporting a potential link between PLRS and immune microenvironment remodeling in CRC. These findings suggest that PLRS is associated with the immune microenvironment landscape of CRC and that high-PLRS tumors may exhibit a more complex tumor microenvironment with abundant non-tumor cells, which could potentially contribute to immunosuppression and reduced treatment sensitivity. Given the critical role of the immune microenvironment in CRC progression, the association of PLRS with these features may provide insights into potential immunotherapy-related mechanisms; however, these observations are correlative and require validation in CRC-specific immunotherapy cohorts before any clinical inference can be drawn.

Single-cell analysis of tumor-immune cell communication revealed significant differences between the high- and low-PLRS groups. In the outgoing direction, the NECTIN pathway exhibited a higher communication intensity in the high-PLRS group. As a cell adhesion molecule, NECTIN mediates immune-tumor cell interactions [26] and is overexpressed in multiple tumors [27]. The enhanced NECTIN-dependent crosstalk in high-PLRS groups may contribute to immune cell activation, tumor cell proliferation, migration, and immunosuppression, potentially offering a partial explanation for the poor prognosis of these patients. Clinically, the NECTIN pathway warrants further investigation as a potential therapeutic target for high-risk CRC patients identified by the PLRS model. In the incoming direction, the EPHA pathway was more active in the high-PLRS group. EPHA receptors regulate cell proliferation, migration, and signaling. EPHA6 was identified as a CRC driver gene in African Americans [28]. Additionally, high-PLRS cells showed distinct communication patterns with macrophages, monocytes, fibroblasts, dendritic cells, and Pro-B-cell-CD34+ cells, suggesting that PL-gene signatures may modulate the crosstalk between immune-, hematopoietic-, and inflammation-related cells in the CRC microenvironment. Platelet-derived extracellular vesicles have been reported to mediate platelet–tumor communication and to regulate malignant behaviors and the tumor microenvironment [29]. These findings shed light on the transcriptional programs associated with platelet-related signatures in CRC progression and may offer potential clues for identifying candidate therapeutic targets for high-risk patients.

However, these single-cell findings should be interpreted with caution due to several limitations. First, due to the sparse nature of scRNA-seq data, PLRS could only be calculated for malignant epithelial cells with at least one detectable feature gene (n = 44,353 of 46,915). This filtering step may introduce selection bias by enriching for cells with higher expression of the signature genes, and single-cell PLRS estimates should therefore be considered exploratory. Second, PLRS is a weighted sum of six gene expression values; when only a subset of genes is detected, undetected genes contribute zero to the score, which may reduce reliability in cells with low detection rates. Third, the cell–cell communication analysis was based on inferred ligand–receptor interactions and does not directly demonstrate functional signaling. Future studies using dropout-aware scoring methods (e.g., UCell) on the full malignant cell population, combined with functional validation, are needed to confirm the robustness of these observations.

Drug sensitivity analysis highlighted the potential of the PLRS model to guide personalized chemotherapy. In the GDSC dataset, the IC50 values of Dasatinib and Sapitinib positively correlated with PLRS, indicating greater resistance in the high-PLRS group, whereas the IC50 values of Daporinad and Tozasertib negatively correlated with PLRS, suggesting a higher sensitivity in the high-PLRS group. Dasatinib is clinically used for CRC treatment [30] and shows increased efficacy in high-risk populations [31], whereas Sapitinib overcomes ABCB1 transporter-mediated multidrug resistance in CRC by enhancing anticancer drug accumulation [32]. There low-PLRS patients may benefit more from Dasatinib or Sapitinib treatments. Daporinad, a novel nicotinamide phosphoribosyltransferase inhibitor with antitumor and antiangiogenic activities [33], and Tozasertib, which inhibits proliferation, induces cell cycle arrest, and reduces tumor angiogenesis across multiple tumor types [34] (with superior efficacy against small intestine adenocarcinoma cells compared to conventional drugs) [35], have not been fully validated in CRC. Our findings indicate that high-PLRS patients may be more sensitive to these two agents, providing a rationale for future clinical trials to evaluate their efficacy in PLRS-stratified CRC cohorts.

In the CCLE dataset, 12 drugs (Afatinib, Alectinib, Anastrozole, Erlotinib, LGK974, Lapatinib, Ibrutinib, Gefitinib, OSI-027, Osimertinib, Sunitinib, and TAF1-5496) showed positive correlations between IC50 values and PLRS, whereas Trametinib showed a negative correlation. These drugs target specific tumor-related genes or proteins to inhibit tumor cell proliferation, survival, and metastasis. For example, Alectinib [36] and Erlotinib [37] inhibit specific oncogenic activity in tumor cells to suppress tumor growth. LGK974 [38] and OSI-027 [39], inhibit signaling pathways, such as Wnt and PI3K, to block tumor cell survival and proliferation. However, because the CCLE dataset is derived from cell lines that do not fully recapitulate the tumor microenvironment, heterogeneity, or systemic immune responses in patients, future prospective clinical trials are needed to validate the predictive value of the PLRS model for therapeutic responses to these drugs in patients with CRC. In this context, recent advances in semiconductor-based biomedical technologies—including point-of-care diagnostic platforms and bio-integrated sensor systems—may facilitate the future translation of biomarker-based models such as PLRS into clinically deployable tools for rapid and cost-effective risk stratification [40].

However, this study has several limitations. First, the PLRS model was developed and validated using public datasets (TCGA and GEO), lacking prospective clinical cohort validation, and it does not integrate clinical parameters (e.g., platelet count) or multi-omics data. Second, experimental validation was confined to tissue and cellular levels without in vivo animal models; moreover, qRT-PCR was limited to one CRC cell line (RKO) and the CCD-18Co fibroblast control, which does not represent normal colonic epithelium. *CA2* was not tested due to primer-design failure, and *TTYH3* showed no significant difference, so the experimental results provide only partial support for the PLRS gene signature, and validation in normal colonic epithelial cells is warranted. Third, drug sensitivity associations were derived from GDSC and CCLE cell line databases and require validation in clinical cohorts, as discussed above.

Future work should address these gaps by conducting prospective validations in real-world settings, developing animal models for functional analysis of key genes, integrating multidimensional data to optimize the PLRS, and validating the signature in normal colonic epithelial cells and CRC-specific clinical cohorts.

## 4. Materials and Methods

### 4.1. Data Collection

CRC tissue and single-cell sequencing data were used in this study. The tissue data comprised a PL-genes sequencing dataset (GSE183635), along with training and validation datasets. The training dataset consisted of TCGA-COAD and TCGA-READ datasets of transcriptome, mutation, copy number variation (CNV), and clinical information from Xena (https://xenabrowser.net/, accessed on 12 October 2022). The validation dataset (GSE39582) comprised transcriptome and clinical data. The GSE178341 dataset was used for single-cell data. All GSE datasets were downloaded from the NCBI database (https://www.ncbi.nlm.nih.gov/geo/, accessed on 14 October 2022) using the “getGEO” function from the “GEOquery” package (v2.64.2).

### 4.2. Data Overview

The GSE183635 dataset comprised 390 normal controls and 85 CRC samples. TCGA-COAD and TCGA-READ were integrated into the TCGA-COADREAD dataset as “raw counts” using the “recount3” R package (v1.8.0). Batch effects were removed using the “ComBat_seq” function from the “sva” R package (v3.44.0). The integrated TCGA-COADREAD dataset comprised 676 sequencing samples: 622 tumor and 54 normal samples. Among these, 598 samples had complete overall survival information and after excluding samples with incomplete clinical information, 573 tumor samples remained. The GSE39582 dataset contained 585 sequencing samples, including 566 tumor samples and 19 normal samples. After excluding samples with incomplete clinical information, 531 tumor samples were retained (Supplementary Table S1).

The GSE178341 dataset comprised 64 samples from 62 patients, with 71,223 single-cell transcriptome sequencing data points. After sample integration and batch correction, the final dataset comprised 231,698 single cells and 43,282 genes.

### 4.3. Differential Gene Expression Analysis

Differential expression analysis was carried out on counts data from the GSE183635 and TCGA-COADREAD datasets using the “DESeq2” R package (v1.36.0). Differentially expressed genes (DEGs) were filtered using *P_adj_* < 0.05 and |log_2_FoldChange| > 0.58 [41,42].

### 4.4. Prognostic Gene Identification

Cox analysis was conducted on the DEGs using the “survival” R package, and genes with significant prognostic values (*p* < 0.05) were identified.

### 4.5. Machine Learning-Based Platelet-Related Scoring Model

LASSO regression analysis was performed using the cv.glmnet function from the glmnet R package, with family = “cox” for survival data. Ten-fold cross-validation was applied to determine the optimal penalty parameter λ, and lambda.min was selected. Genes with non-zero coefficients at lambda.min were retained in the final model. The platelet-related scoring model, termed PLRScore, was constructed by summing the products of the LASSO regression coefficients and gene expression levels.

$$PLRScore=\sum_{i=1}^{n}expression\;of\;gene\;i*lasoo\;coefficient\;of\;gene\;i$$

### 4.6. Cell Type Annotation of Single Cells

Using the “SingleR” R package (v1.10.0), cell type annotation was performed on single-cell subpopulations based on hierarchical principal component analysis. Default parameters were applied for the “SingleR” function.

### 4.7. Single-Cell CNV Analysis

The “CopyKAT” package(v1.1.0) was used to analyze single-cell CNV profiles. Epithelial cells isolated and annotated from samples were subjected to CNV analysis, with diploid cells, macrophages, monocytes, and Natural Killer cells serving as references. CNVs have been used to estimate the clonal evolution in stem cells. Parameters for “CopyKAT” were set as: id.type = “S”, ngene.chr = 5, win.size = 25, and KS.cut = 0.1.

### 4.8. Discrimination of Malignant and Non-Malignant Cells

Clustering of CNVs from epithelial and normal cells was conducted using the “CopyKAT” R package (v1.1.0). Cells were classified into two categories: epithelial cells clustered with normal cells were defined as non-malignant and those in the other cluster were considered malignant.

### 4.9. Single-Cell Pseudotime Trajectory Analysis

Pseudotime trajectory analysis was performed on the 44,353 malignant epithelial cells using Monocle3. Following standard preprocessing and graph learning with the “learn_graph()” function, high-PLRS cells were designated as the trajectory root based on their association with aggressive tumor phenotypes in bulk analyses. Root principal nodes were identified using “get_earliest_principal_node()”, and pseudotime was calculated for all cells using “order_cells()”. The trajectory was visualized on UMAP embeddings with cells colored by pseudotime, revealing a transcriptional continuum from high- to low-PLRS populations.

### 4.10. Overall Survival and Prognostic Prediction

The samples were divided into high- and low-PLRS groups based on median PLRSs. The Kaplan–Meier method was used to estimate the difference in overall survival between the high- and low-PLRS groups. Furthermore, receiver operating characteristic (ROC) curves and areas under the curve (AUCs) were generated to compare the prognostic abilities of the different models.

### 4.11. Immune Infiltration

The ESTIMATE algorithm was used to describe the infiltration rates of immune and stromal cells. Subsequently, the ssGSEA, Cibersort, and xCell algorithms were then used to analyze the cellular components of the tumor microenvironment.

### 4.12. Cell Communication

The “monocle3” R package (v1.2.9) was used to analyze the transcriptional dynamics of tumor cells, mapping the different states and transcriptional regulatory processes of CRC cells. Additionally, the R packages, “celltalker” (v0.2.0) and “CellChat” (v1.1.3) were used for an in-depth analysis of communication between immune cells and CRC cells. The “celltalker” package was used to specifically identify ligand–receptor pairs between immune and tumor cells in scRNA-seq data.

### 4.13. Correlation Analysis Between PLRS and Drug Sensitivity

Drug sensitivity data for approximately 1000 cancer cell lines were downloaded from the GDSC database (http://www.cancerrxgene.org, accessed on 9 October 2022). The IC50 values of antitumor drugs in cancer cell lines were used as an indicator of drug response. Spearman’s correlation analysis was performed to calculate the correlation between drug sensitivity and PLRS, considering |Rs| > 0.2 (|Rs| represents the absolute value of Spearman’s rank correlation coefficient) and *p* < 0.05 as statistically significant. The same method was applied to the Cancer Cell Line Encyclopedia (CCLE) dataset (https://depmap.org/portal/download/, accessed on 9 October 2022).

### 4.14. Cell Culture and qRT-PCR

Normal human colon fibroblast cell line CCD-18Co (Cell Bank of the Chinese Academy of Sciences, Shanghai, China) and the human colorectal adenocarcinoma cell line RKO (Affiliated Tumor Hospital of Inner Mongolia Medical University, Hohhot, China), were cultured in Dulbecco’s modified Eagle’s medium (DMEM; Cat.No. PM150210; Procell Life Science & Technology Co., Ltd., Wuhan, China) supplemented with 10% fetal bovine serum (FBS; Cat.No. 11011-8611; Zhejiang Tianhang Biotechnology Co., Ltd., Hangzhou, China). Total RNA was extracted from these cell lines using Trizol (RNAiso Plus) reagent (Cat.No. 9108; Takara Bio Inc., Kusatsu, Shiga, Japan), followed by reverse transcription into complementary DNA using a PrimeScript RT Reagent Kit with gDNA Eraser (Cat.No. RR047A; Takara Bio Inc., Kusatsu, Shiga, Japan). qRT-PCR was performed using TB Green Premix Ex Taq II (Tli RNaseH Plus) (Cat.No. RR820A; Takara Bio Inc., Kusatsu, Shiga, Japan) containing SYBR Green I fluorescent dye. The primer sequences are provided in Supplementary Table S2.

### 4.15. Statistical Analysis

Wilcoxon’s test was used for comparative analysis. ROC curves were used to validate the effectiveness of the models. In the TCGA training cohort, patients were dichotomized into high- and low-risk groups based on the median PLRS. This same median value was then locked as a fixed cutoff and directly applied to the external validation cohorts (GSE39582 and GSE183635) without recalibration. Survival curves were plotted using the Kaplan–Meier method for prognostic analysis. The log-rank test was used to determine the significance of differences. To assess whether the PLRS was an independent prognostic factor, a multivariate Cox regression model analysis was conducted, including variables such as age, sex, and stage. All statistical analyses were two-sided, with *p* < 0.05 considered statistically significant.

## 5. Conclusions

A PLRS model was established using six key genes (*CA2*, *GPX4*, *LITAF*, *PLCB4*, *TIMP1*, and *TTYH3*), which demonstrated modest but significant prognostic efficacy. The integration of scRNA-seq data further elucidated the biological mechanisms underlying PLRS, revealing that high and low PLRSs correlated with distinct immune microenvironment landscapes, tumor-immune cell communication patterns (e.g., NECTIN/EPHA pathways), and potential responses to immune checkpoint inhibitors. The critical roles of PLRS and its constituent genes in CRC progression were established. Additionally, the PLRS model showed potential associations with sensitivity to several chemotherapeutic and targeted agents (e.g., Dasatinib, Sapitinib, and Daporinad) based on GDSC and CCLE cell line data. However, we acknowledge that these predictions are derived from preclinical models and do not directly establish clinical treatment response. Therefore, prospective clinical studies are warranted to evaluate the utility of PLRS in guiding individualized therapy.

## Data Availability

The TCGA-COAD and TCGA-READ datasets were collected from Xena (https://xenabrowser.net/, accessed on 12 September 2022). GSE183635, GSE178341 and GSE39582 were downloaded from the NCBI database.

## Supplementary Materials

The following supporting information can be downloaded at: https://www.mdpi.com/article/10.3390/ijms27188312/s1.
