# Supplementary material for: Platelet-Related Signature in Colorectal Cancer: Prognosis and Therapy Guidance
Source: Int J Mol Sci. 2026 Sep 18;27(18):8312. doi: 10.3390/ijms27188312 (PMC13607224; doi:10.3390/ijms27188312)
Supplement: Supplementary file 1 [file ijms-27-08312-s001.zip › ijms-4521825-supplementary.pdf]

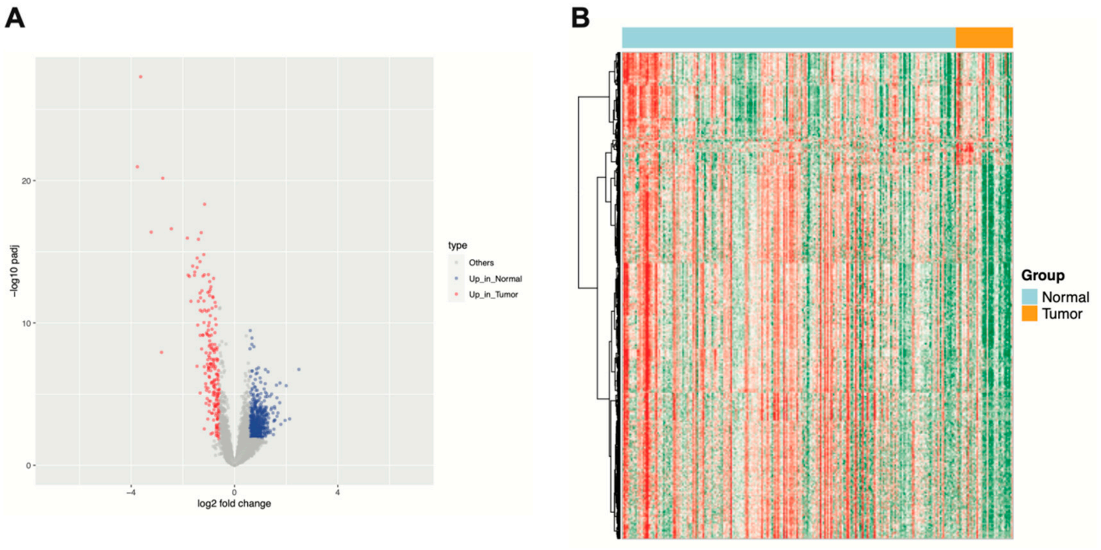

Supplementary Figure S1 Differential expression of platelet-related factors between CRC samples and normal samples. A Volcano plot of differentially expressed platelet-related factors. B Heatmap of differentially expressed platelet-related factors

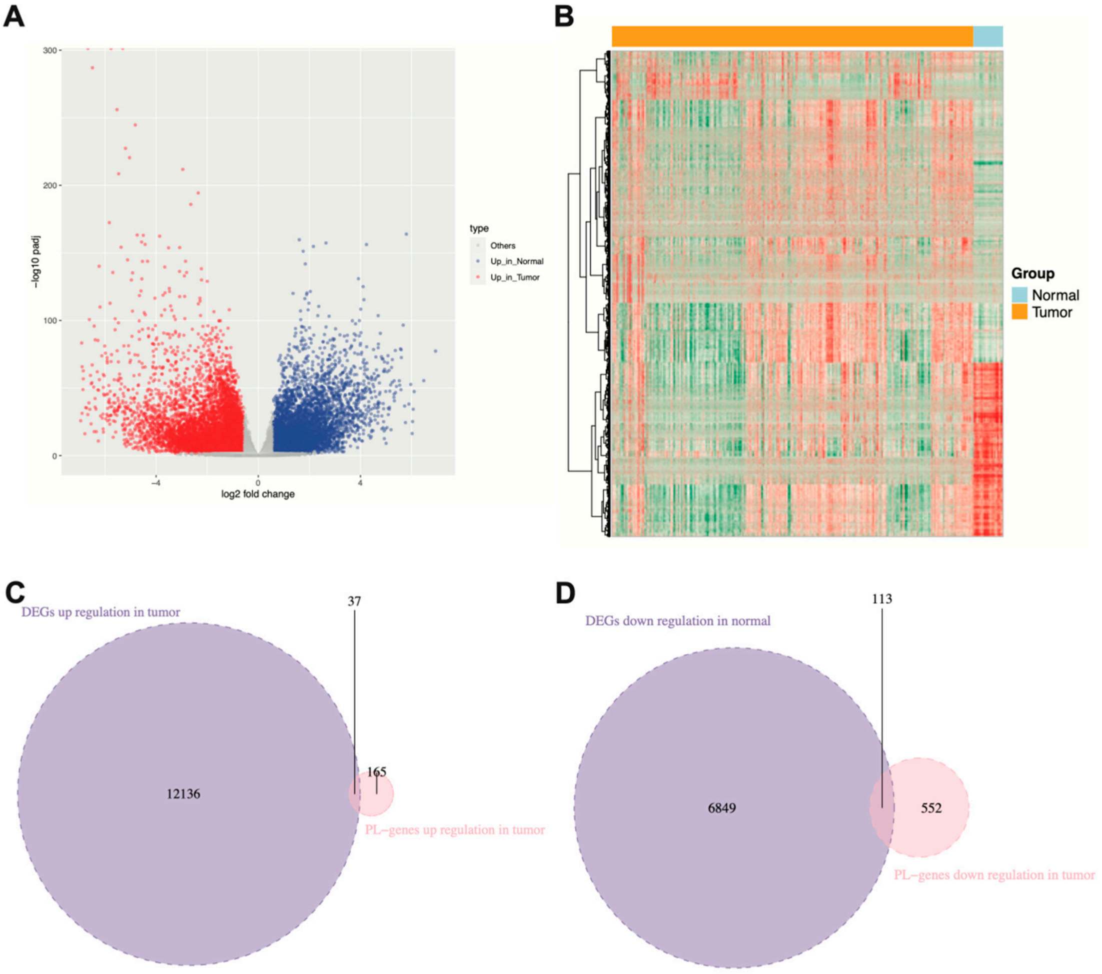

Supplementary Figure S2 Differential expression gene and PL-gene selection between normal samples and tumor samples. **A** Volcano plot of differentially expressed genes. **B** Heatmap of differentially expressed genes. **C** Venn diagram showing upregulated genes in tumor and platelet-related factors. **D** Venn diagram showing upregulated genes in normal samples and platelet-related factors

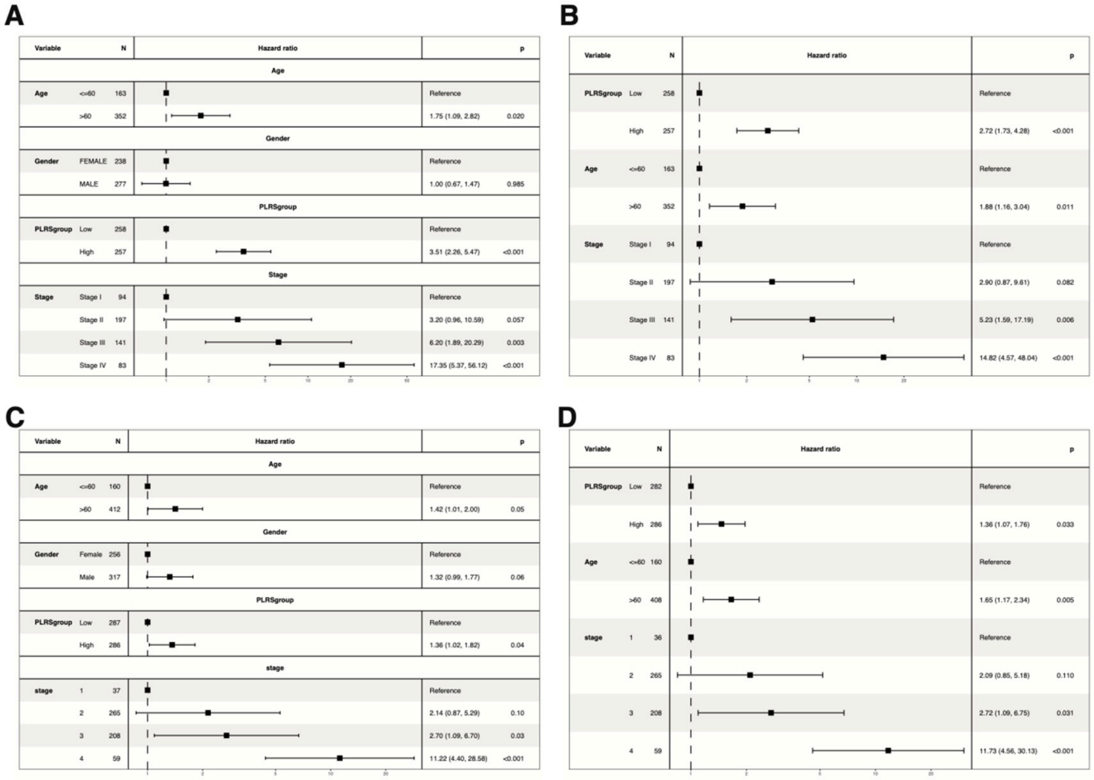

Supplementary Figure S3 Univariate and multivariate Cox analyses. **A** Univariate Cox analysis in TCGA dataset. **B** Multivariate Cox analysis in TCGA dataset. **C** Univariate Cox analysis in GSE39582 dataset. **D** Multivariate Cox analysis in GSE39582 dataset

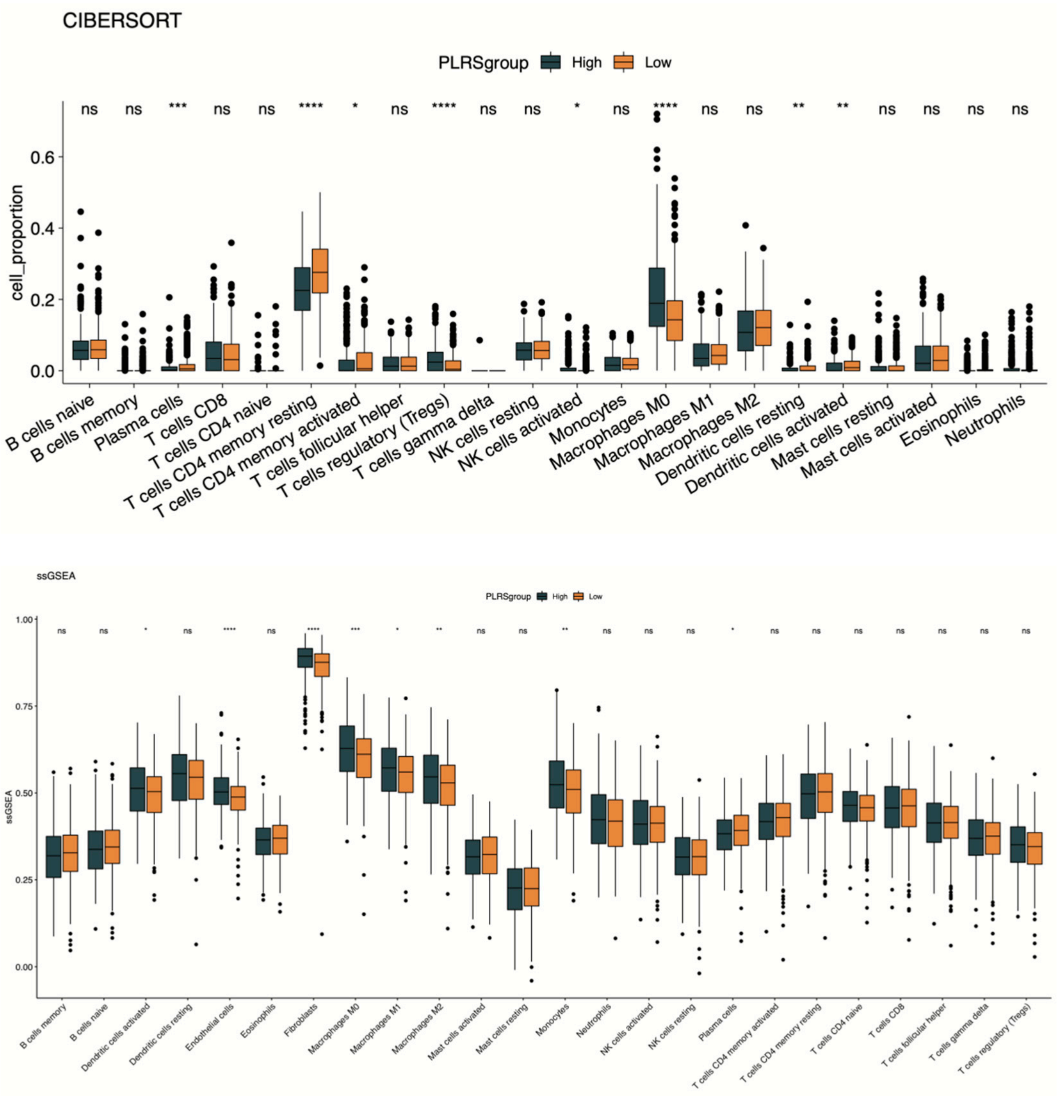

Supplementary Figure S4 The immune landscape between the high PLRS and low PLRS groups

Supplementary Table S1 Colorectal Cancer Tissue Data Information

		TCGA	GSE39582
Status	alive	462	358
	Dead	111	173
Age	<=60	185	152
	>60	388	379
Gender	Male	310	287
	Female	263	244
T	T1	19	11
	T2	103	43
	T3	391	360
	T4	60	117

N	N0	327	294
	N1	141	133
	N2	105	98
	N3	-	6
M	M0	440	469
	M1	84	60
	MX	49	2
Stage	Stage I	106	31
	Stage II	211	251
	Stage III	170	190
	Stage IV	86	59

Supplementary Table S2 qRT-PCR Primer Sequences

Gene Name	Upstream Primer	Downstream Primer
TIMP1	5'-GTTGTTGCTGTGGCTGATAG-3'	5'-TGTGGGACCTGTGGAAGTA-3'
LITAF	5'-ATGTCGGTTCCAGGACCTTAC-3'	5'-TACGAAGGAGGATTCATGCCC-3'
TTYH3	5'-TGCTGAATGGCACGGAGGTGAA-3'	5'-CTCCACGCCGTCATAGCAGAAG-3'
GPX4	5'-AAGTCCTAGGAAACGCCCG-3'	5'-CATCGCGGGATGCACACAAG-3'
PLCB4	5'-GCCCATTACTTCATCAGTTCCT-3'	5'-TACACATTGCTTTTCCGTGAGT-3'
